# Scopolamine fatal outcome in an inmate after buscopan® smoking

**DOI:** 10.1007/s00414-021-02583-2

**Published:** 2021-04-23

**Authors:** Sabina Strano-Rossi, Serena Mestria, Giorgio Bolino, Matteo Polacco, Simone Grassi, Antonio Oliva

**Affiliations:** 1 Section of Legal Medicine, Department of Health Surveillance and Bioethics, Università Cattolica del Sacro Cuore, Largo F. Vito 1, 00168 Rome, Italy; 2 Unit of Forensic Pathology, SAIMLAL Department, Sapienza University of Rome, Rome, Italy

**Keywords:** Scopolamine, Scopolamine N-butylbromide, Forensic toxicology, Poisoning, PMCT

## Abstract

Scopolamine is an alkaloid which acts as competitive antagonists to acetylcholine at central and peripheral muscarinic receptors. We report the case of a 41-year-old male convict with a 27-year history of cannabis abuse who suddenly died in the bed of his cell after having smoked buscopan® tablets. Since both abuse of substances and recent physical assaults had been reported, we opted for a comprehensive approach (post-mortem computed tomography CT (PMCT), full forensic autopsy, and toxicology testing) to determine which was the cause of the death. Virtopsy found significant cerebral edema and lungs edema that were confirmed at the autopsy and at the histopathological examination. Scopolamine was detected in peripheral blood at the toxic concentration of 14 ng/mL in blood and at 263 ng/mL in urine, and scopolamine butyl bromide at 17 ng/mL in blood and 90 ng/mL in urine. Quetiapine, mirtazapine, lorazepam, diazepam, and metabolites and valproate were also detected (at therapeutic concentrations). Inmates, especially when they have a history of drug abuse, are at risk to use any substance they can find for recreational purposes. In prisons, active surveillance on the management and assumption of prescribed drugs could avoid fatal acute intoxication.

## Introduction

Scopolamine is one of the main alkaloids, together with atropine, contained in many plants from the *Solanaceae* family such as *Datura* species, *Hyoscyamus*, and *Atropa belladonna*. Tropane *Solanaceae* alkaloids are contained mainly in the seeds, flowers, and leaves. They have strong anticholinergic activity and act as competitive antagonists to acetylcholine at central and peripheral muscarinic receptors. They penetrate the blood–brain barrier and bind to acetylcholine receptors in the cortex and subcortical regions of the brain. At low doses, tropane alkaloids cause peripheral anticholinergic signs including decreased salivation and sweating, mydriasis, loss of accommodation, increased pulse rate, and sinus tachycardia. As the dose increases, ileus, urinary retention, and elevated temperature occur followed by the beginning of central nervous system (CNS) effects (confusion, agitation). High doses of *belladonna* alkaloids produce delirium, hallucination, and coma, with higher activity of scopolamine compared to atropine [1].

Buscopan® is a pharmaceutical product containing the quaternary ammonium derivative scopolamine N-butylbromide. It acts primarily on parasympathetic ganglia in the walls of the viscera, with antispasmodic action on smooth muscle in the gastrointestinal, biliary, and urinary tracts. It is indicated in the symptomatic treatment of spastic-painful manifestations of the gastroenteric and genitourinary tract and is produced as tablets, suppositories, and injectable solutions [2]. Overdose symptoms include tachycardia, hypotension, drowsiness, urinary retention, dry mouth, skin redness, inhibition of gastrointestinal motility, transient visual disturbances, and respiratory paralysis. The effects can be increased by concomitant use of other anticholinergic or central acting drugs. Scopolamine N-butylbromide does not pass the blood–brain barrier. No scopolamine is detected in biological fluids after buscopan intake [3].

The formation of scopolamine from its N-butyl bromide after treatment at high temperature via microwaves or after smoking has been described [3, 4]. The habit of smoking buscopan® tablets by inmates, due to its hallucinogenic effects, was described in previous reports, and the intoxication symptoms were attributed to scopolamine, formed by the pyrolysis of the N-butylbromide derivative, as demonstrated by the experimental study [3, 5].

We report the case of a male convict who suddenly died in the bed of his cell. Since both abuse of substances (buscopan® tablets smoking) and recent physical assaults had been reported, we opted for a comprehensive approach (post-mortem CT (PMCT), full forensic autopsy, and toxicology testing) to determine which was the cause of the death.

## Case presentation and PMCT/autopsy findings

**Fig. 1:**
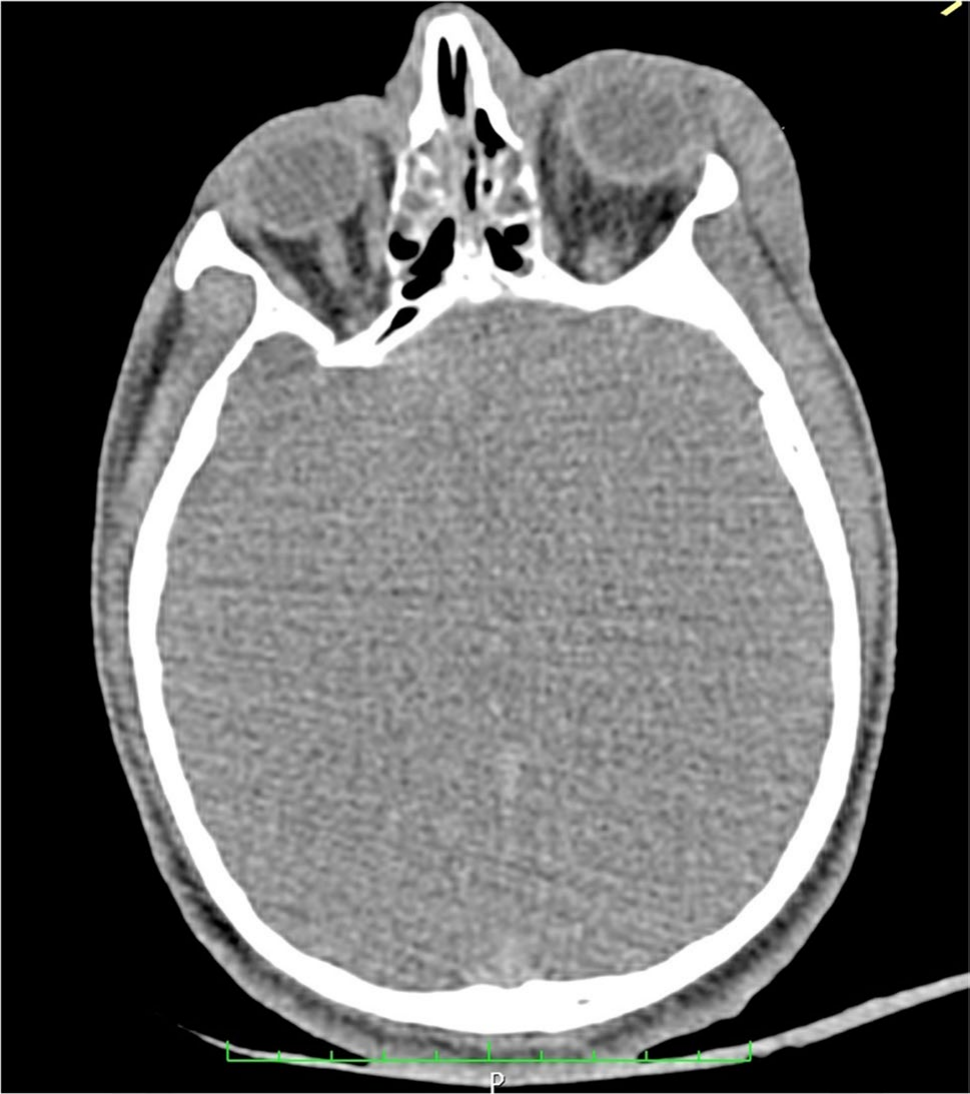
CT image showing massive cerebral edema

**Fig. 2:**
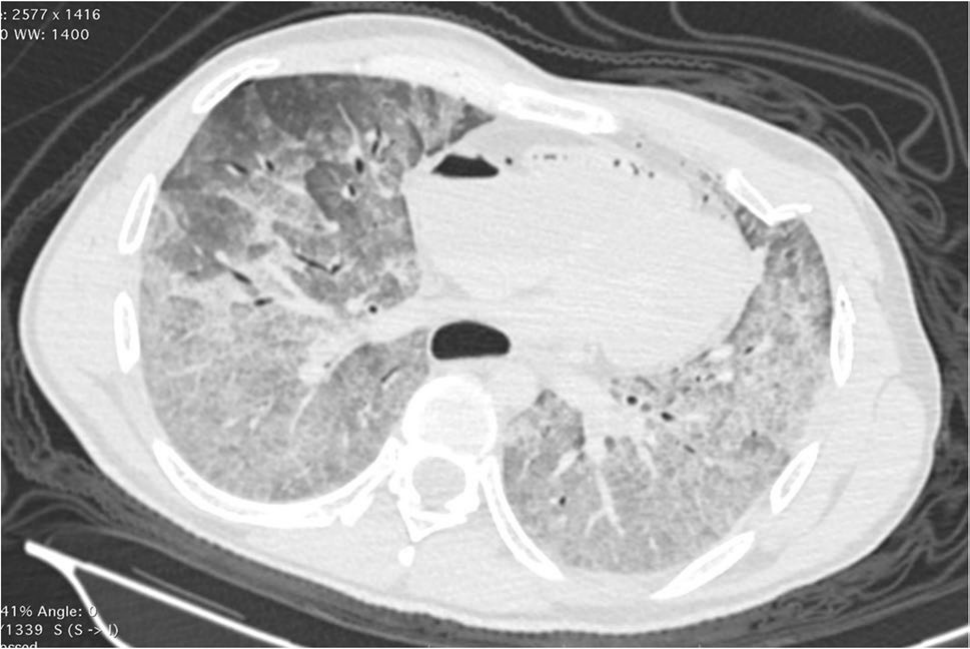
CT image showing massive edema of the lungs

A 41-year-old male convict (height: 165 cm; weight: 72 kg; body mass index: 26,4) with a 27-year history of cannabis abuse was found dead in the bed of his cell. Other inmates reported that he had recently smoked an undefined number of buscopan® tablets, obtained by a cellmate. Depakin® (natrium valproate), seroquel® (quetiapine), and remeron® (mirtazapine) tablets were found on the floor of his cell. Four days and the day before his death, he had been assaulted, suffering multiple bruises on face, chest, epigastric area and lower limbs, a wound above the left eyebrow, and fractures of six left ribs (from the second to the seventh), and seven right ribs (from the second to the eight). A public prosecutor requested a full forensic autopsy to evaluate whether the death had been caused by the assaults or by a drug intoxication. We performed a CT scan using a Somatom Sensation 16 CT scanner (Siemens®, Munich, Germany). We adopted these parameters: 140 kVp, 160 mAs, 24-mm feed/rotation, 1-mm slice collimation, 1-mm slice width, and 10–30-40–70-80 reconstruction kernel. After data processing, axial, coronal and sagittal two- dimensional (2D) reconstructions and a 3D-reconstruction with volume rendering (VR) and shaded surface display (SSD) were performed. Virtopsy found significant cerebral edema (**Fig. ****1**) and lungs edema (**Fig. ****2**).

Virtopsy also confirmed the fractures caused by the assaults and did not find any possible cause of the death. Post-mortem nasal and pharyngeal swabs were performed in accordance with current guidelines [6] and excluded a SARS-CoV-2 infection. Hence, we performed a full forensic autopsy and, then, a histopathologic examination of the organs, which confirmed the PMCT findings.

## Toxicological analyses

### Materials and methods

#### Chemicals and reagents

Scopolamine hydrochloride, chloroform, ethyl acetate, methanol, formic acid, ammonium formate, bis-trimethylsylil trifluoroacetamide (BSTFA), and ultrapure water were supplied by Sigma-Aldrich (Milan, Italy). Scopolamine butyl bromide and sodium valproate were obtained by Sanofi (Milan, Italy) as 20 and 200 mg/mL solutions, respectively. Zolpidem-D6 (Internal standard) was purchased from Lipomed AG (Arlesheim, Switzerland) as 0.1 µg/mL solution. One µg/mL solutions were prepared and used as working solutions to prepare the calibration curves.

#### Samples preparation

Urine samples were analyzed by LC–MS/MS using a dilute and shoot approach. One hundred microliter of urine were diluted with 400 μL of 0.1% aqueous formic acid, added with a mixture of deuterated internal standards (0.1 µg/mL) and directly injected in the UHPLC-MS/MS system. For the analysis of benzodiazepines another aliquot of sample prepared as described above was preliminary submitted to enzymatic hydrolysis by β-glucuronidase in acidic medium.

Peripheral blood samples (0.5 mL) were analyzed and quantified by LC–MS/MS after DLLME (dispersive liquid/liquid microextraction) or after deproteinisation with 0.5 mL of methanol and ultracentrifugation (for scopolamine butylbromide). Detailed analytical conditions for the analysis of blood samples and methods validations are reported elsewhere [7, 8].

Valproic acid determination was performed by GC–MS after liquid/liquid extraction of 2 mL of samples in acidic conditions with ethyl acetate. The extracts were collected, evaporated to dryness, and derivatized with BSTFA (50 μL). One microliter of the extract was injected into the GC port. Calibration curve was in the range 10–100 g/L.

#### Instruments

##### LC–MS/MS

The UHPLC instrument was an Agilent 1290 Infinity system: binary pump with integrated vacuum degasser, high-performance well-plate autosampler, and thermostated column compartment modules. The detection system was an Agilent 6460 triple quadrupole mass spectrometer (Agilent Technologies, Santa Clara, CA, USA) with a Jet-Stream electrospray ionization source in positive mode. Instrumental parameters were set as follows: gas temp 350 °C, gas flow 9 mL/min, nebulizer 40 psi, column flow 0.4 mL/min, sheath gas heater 400 °C, sheath gas flow 11 psi, and capillary voltage 4000 V. Chromatographic separation was achieved using a superficially porous Raptor C18 column (2.7 μm, 100 × 2.1 mm from Restek, Milan, Italy), mobile phase A: H_2_O 0.1% HCOOH/0.1% ammonium formate and B: MeOH 0.1% HCOOH, with a gradient from 10 to 90% B in 14 min. MRM transitions for the detected drugs were the following: Scopolamine 304 156, 138, 103; Scopolamine butyl bromide 360 194, 138; Quetiapine 384 279, 253, 221; Diazepam 285 257, 222, 154; Nordiazepam 271 208, 165, 140; OH-triazolam 359 331, 239, 176; Triazolam 343 315, 308, 239; Oxazepam 287 269, 242, 163; Lorazepam 321 275, 229, 163 Temazepam 301 255, 177, 199; Mirtazapine 266 209, 195, 72.

MRM transitions for the deuterated standards were the following: Zolpidem D6 314 235, 263; Alprazolam D5 314 286, 210; Diazepam D5 290 154, 227; Nordiazepam D5 276 140, 165; Triazolam D4 347 312, 319; Oxazepam D5 292 246, 109 Lorazepam D4 325 279, 233. Underlined ions were used for quantitation.

The limits of detection were 0.2 ng/mL for scopolamine and 1 ng/mL for scopolamine butyl bromide, and the limits of quantitation (LOQ) were 0.5 ng/mL for scopolamine and 2 ng/mL for scopolamine butyl bromide. The method was linear in the range from the LOQ to 200 ng/mL for both the substances, giving correlation coefficients of the curves > 0.99 for scopolamine and > 0.98 for scopolamine butyl bromide. The accuracy, intended as %E, was < 15% for scopolamine at 2 and 50 ng/mL and < 20% at 2 ng/mL and < 15% at 50 ng/mL for scopolamine butyl bromide. The validation parameters for the other analytes detected are reported elsewhere [5].

##### GC–MS

The GC–MS system was an Agilent 7890 gas chromatograph coupled to an Agilent 5975c quadrupole mass detector (Agilent Technologies Italia, Milan, Italy) operating at 70 eV in electron ionization mode. The chromatographic conditions were the following: J&W 5% phenyl-methylsilicone capillary column (17 m × 0.2 mm i.d., 0.33-µm film thickness, CPS Analitica, Milan, Italy). Helium was used as carrier gas at a constant flow of 1 mL/min. The oven temperature was held at 75 °C for 2 min, increased to 270 °C at 15 °C/min, and increased to 310 °C at 50 °C/min (held for 4 min). The injection port was set at 270 °C in splitless mode. The mass detector was operated in SIM mode for valproic acid-TMS determination (acquired ions are *m/z* 174, 201, 191, and 145 for valproic acid and *m/z* 206 and 233 for GHB-D6, I.S.).

## Toxicological findings

**Fig. 3:**
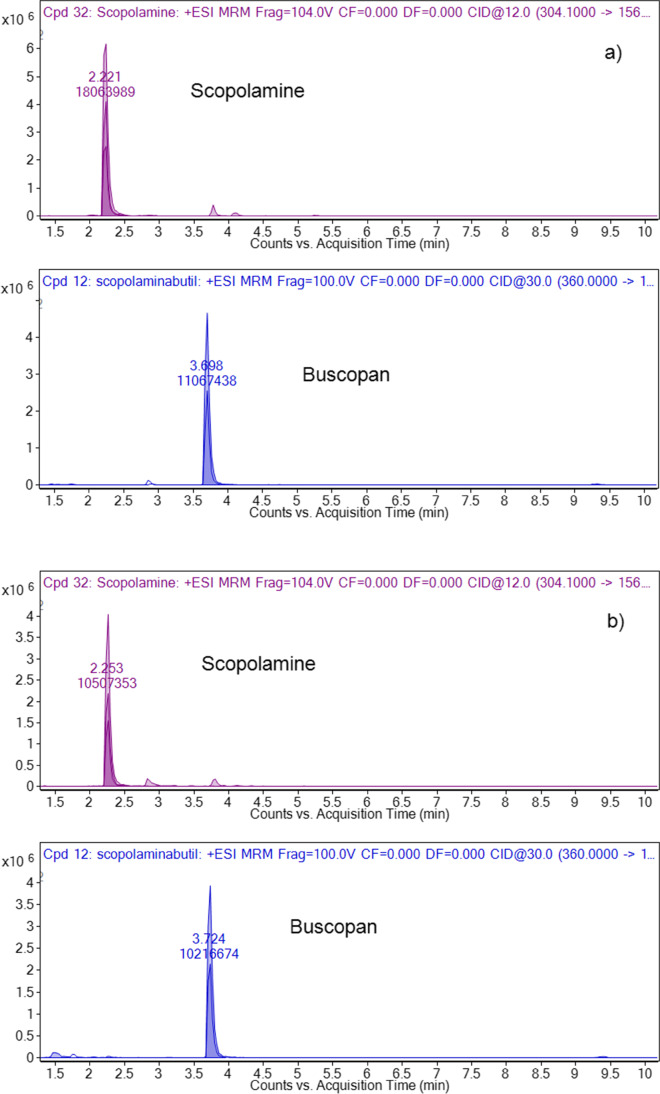
Extracted ionic chromatograms of scopolamine and scopolamine butyl bromide characteristic transitions in the urine of the deceased (**a**) and in a reference positive sample (**b**)

Toxicological analyses allowed the detection of different drugs in the biological samples of the deceased. Scopolamine was detected at the toxic concentration of 14 ng/mL in blood and at 263 ng/mL in urine, and scopolamine butyl bromide at 17 ng/mL in blood and 90 ng/mL in urine. The other drugs identified included benzodiazepines (diazepam and active metabolites nordiazepam, oxazepam and temazepam, triazolam, lorazepam), antipsychotic drugs (quetiapine), and antidepressant drugs (mirtazapine and valproic acid). All of them were in therapeutic or subtherapeutic concentrations. The analytical findings are summarized in Table 1. The extracted ion chromatograms of characteristic transitions of scopolamine and scopolamine N-butylbromide in the urine of the victim and in a reference positive sample at 100 ng/mL are depicted in **Fig. ****3**. The anti-inflammatory drug ibuprofen was also detected in the blood and urine of the deceased.

**Table 1:** Substances detected in blood and urine from the deceased subject (ng/mL)

Substance	Peripheral blood	Urine
Scopolamine N-butylbromide (buscopan®)	17	90
Scopolamine	14	263
Quetiapine	247	234
Mirtazapine	26	165
Triazolam	7	6
Lorazepam	68	609
Diazepam	103	Below LOQ
Nordiazepam	190	1549
Temazepam	35	Below LOQ
Oxazepam	67	Below LOQ
Valproic Acid	25,000	12,000

## Discussion

We report the case of a convict who suddenly died in his cell. Since a traumatic cause of death was suspected because of two recent assaults, we performed a PMCT and then a full forensic autopsy, as recommended by the scientific literature [9, 10]. Both PMCT and autopsy did not find any possible cause of the death, only revealing cerebral and lungs edema. Since a recent abuse of substances (buscopan® tablets), valproic acid (depakin®) (natrium valproate), seroquel® (quetiapine), and remeron® (mirtazapine) was suspected, we performed toxicological testing that found significant levels of scopolamine and scopolamine butyl bromide in blood and urine, thus indicating a fatal acute intoxication by scopolamine. The concentrations of quetiapine, mirtazapine, triazolam, hydroxy-triazolam, lorazepam, diazepam, and metabolites and valproic acid were in the therapeutic ranges [11].

Scopolamine and atropine intake are generally performed by ingestion of an infusion of the leaves or of the seeds of some plants of the S*olanaceae* family, such as *Datura stramonium*, *Atropa belladonna*, or *Mandragora officinalis*. This way of administration causes intoxications with hallucinations, which are the desired effect. Severe complications are often associated with this habit, as anticholinergic and psychotic-like symptoms, but they rarely have a lethal outcome. Cases of paralysis and convulsions following ingestion of flowers of plants of the genus *Datura* are reported [1, 12, 13]. Severe poisonings and at least one death after the ingestion of *Datura stramonium* seeds or wheat contaminated with seeds were reported. In cases of severe non-fatal intoxications, blood concentrations ranging from 0.5 to 0.8 ng/mL 3 h after the intake were reported; in one case of hypoxemia and coma, blood and urine concentrations were 1.2 and 132 ng/mL, respectively [14]. In non-fatal, severe intoxications occurred in 21 adults after drinking a tea containing scopolamine, an average serum value of 13 ng/mL was detected, similarly to the present case [13]. Scopolamine was detected in serum at 2.0 and 0.5 ng/mL in two subjects that ingested scopolamine obtained by the pyrolysis of buscopan in a microwave oven, while urinary concentrations were, respectively, 83 and 20 ng/mL after more than 12 h from the ingestion. One of them presented uncontrolled, clonic movements, non-sensical speech, flushed and dry skin, symmetric mydriasis, and intermittent aggressive behavior [3].

Unlike other cases reported in the literature, in which the way of administration was ingestion, in the present case, scopolamine was administered by smoking crushed buscopan® tablets. The pyrolytic transformation of scopolamine N-butylbromide into scopolamine and its inhalation presumably caused the rapid absorption of a toxic amount of the drug, compatible with a lethal intoxication, as for the high concentration of scopolamine in peripheral blood (14 ng/mL) and in urine (263 ng/mL). After therapeutic use, scopolamine concentrations are in fact reported between 0.1 and 0.3 ng/mL while are considered toxic/lethal concentrations from 1.2 ng/mL [11]. Nevertheless, in a fatal scopolamine intoxication, the drug concentration in blood reached 1890 ng/mL [14].

In the described case, the presence of other central acting drugs such as valproate, benzodiazepines, mirtazapine, and quetiapine, although in therapeutic concentrations, may have contributed to the fatal outcome by a central depression and alteration of the cardiac rhythm.

The misuse of an easily available over the counter drug, considered as essential medicine by WHO, being one of the *efficacious*,* safe*, and* cost-effective medicines for priority conditions* [15], must be carefully considered as a possible health risk, especially for inmates. In this population, the search of psychotropic effects by off-label use of psychotropic medicines, which can be more easily available in jail, may happen [16, 17].

## Conclusions

In this paper, we report the death of a convict caused by an acute intoxication of scopolamine in association to valproate, benzodiazepines, mirtazapine, and quetiapine (in therapeutic concentrations). Inmates, especially when they have a history of drug abuse, are at risk of using any substance they can find for recreational purposes [17]. Therefore, in prisons, the active surveillance on the management and assumption of prescribed drugs could avoid fatal acute intoxication.

## Data Availability

Access to data is possible on reasonable request.
